# Verbal working memory and inhibition‐concentration in adults with cochlear implants

**DOI:** 10.1002/lio2.90

**Published:** 2017-07-19

**Authors:** Aaron C. Moberly, Derek M. Houston, Michael S. Harris, Oliver F. Adunka, Irina Castellanos

**Affiliations:** ^1^ Department of Otolaryngology The Ohio State University Wexner Medical Center

**Keywords:** Cochlear implants, sensorineural hearing loss, speech perception, inhibition‐concentration, verbal working memory

## Abstract

**Objectives:**

Neurocognitive functions contribute to speech recognition in postlingual adults with cochlear implants (CIs). In particular, better verbal working memory (WM) on modality‐specific (auditory) WM tasks predicts better speech recognition. It remains unclear, however, whether this association can be attributed to basic underlying modality‐general neurocognitive functions, or whether it is solely a result of the degraded nature of auditory signals delivered by the CI. Three hypotheses were tested: 1) Both modality‐specific and modality‐general tasks of verbal WM would predict scores of sentence recognition in speech‐shaped noise; 2) Basic modality‐general neurocognitive functions of controlled fluency and inhibition‐concentration would predict both modality‐specific and modality‐general verbal WM; and 3) Scores on both tasks of verbal WM would mediate the effects of more basic neurocognitive functions on sentence recognition.

**Study Design:**

Cross‐sectional study of 30 postlingual adults with CIs and thirty age‐matched normal‐hearing (NH) controls.

**Materials and Methods:**

Participants were tested for sentence recognition in speech‐shaped noise, along with verbal WM using a modality‐general task (Reading Span) and an auditory modality‐specific task (Listening Span). Participants were also assessed for controlled fluency and inhibition‐concentration abilities.

**Results:**

For CI users only, Listening Span scores predicted sentence recognition, and Listening Span scores mediated the effects of inhibition‐concentration on speech recognition. Scores on Reading Span were not related to sentence recognition for either group.

**Conclusion:**

Inhibition‐concentration skills play an important role in CI users' sentence recognition skills, with effects mediated by modality‐specific verbal WM. Further studies will examine inhibition‐concentration and WM skills as novel targets for clinical intervention.

**Level of Evidence:**

4.

## INTRODUCTION

Speech recognition outcomes among adults with acquired sensorineural hearing loss who receive cochlear implants (CIs) are not solely a result of factors relating to the device and the condition of the peripheral auditory system (i.e., “bottom‐up factors”). Rather, linguistic knowledge and basic modality‐general neurocognitive functions–sensory processing functions that are not specific to the modality of input (e.g., visual versus auditory)–likely influence speech recognition outcomes as well.^1^, ^2^, ^3^, ^4^, ^5^ The influence of these “top‐down” factors on robust spoken language recognition is believed to be grounded in the listener's ability to use these previously developed skills (prior to hearing loss) to make sense of the CI's incoming degraded speech signal.^6^

One neurocognitive function thought to be particularly relevant to success in speech recognition for individuals with hearing loss is verbal working memory (WM).^5^, ^7^, ^8^ WM in general is commonly defined as a limited‐capacity, temporary storage mechanism for holding information.^9^, ^10^, ^11^ This mechanism serves a vital role in temporarily maintaining information for further processing, such as during the process of recognizing and comprehending spoken language. Most models share the property of dual mechanisms: a short‐term encoding/storage component, and a processing component.^11^, ^12^, ^13^ Tasks that challenge the storage and processing components simultaneously are considered as measures of an individual's overall verbal WM capacity. Empirical support for a role of verbal WM in speech recognition comes from studies of adults with lesser degrees of hearing loss, and it has been demonstrated that a large verbal WM capacity facilitates the use of linguistic information during the process of speech recognition.^1^, ^14^ Few studies have examined the topic of verbal WM or other neurocognitive processing and speech recognition in adult CI users.

To avoid the confounding factor of audibility in studies of individuals with hearing loss, a commonly used measure of verbal WM capacity that is the Reading Span (RSpan) task, during which sets of sentences are presented orthographically.^2^, ^11^, ^15^, ^16^ To complete the task, the participant must indicate whether each sentence makes sense or not, and then is asked to recall, in serial order, the first or last word of each sentence they read. The number of correct words retained in memory serves as a well‐validated measure of verbal WM capacity. Because the RSpan task eliminates the factor of audibility, it serves as a modality‐general measure of verbal WM capacity and has been found to relate to speech recognition abilities for patients with hearing loss. For example, Arehart et al. identified verbal WM capacity as measured by RSpan as a significant factor in listeners' recognition of sentences in babble processed with frequency compression, accounting for 29% of the variance in recognition scores.^2^ Similarly, Lunner examined 72 elderly patients with mild‐to‐moderate hearing loss and found significant correlations (*r* = 0.4–0.5) between RSpan scores and speech reception thresholds for sentences in modulated noise.^17^

The use of RSpan in adult patients with hearing loss is a modality‐general measure of the participant's total capacity to encode, store, and process words in WM under relatively ideal (visual) presentation of items. However, use of this measure neglects the fact that the verbal WM processing most relevant to speech perception is modality‐specific to auditory input; that is, the listener with hearing loss must encode, store, and process degraded auditory input. Consequently, it is likely that use of RSpan will not be as strongly representative as a modality‐specific auditory task of the verbal WM processing demands required when listening to speech presented auditorily. Instead, for patients with hearing loss listening to spoken language, verbal WM requires encoding of phonological structure accessed from the degraded auditory stimuli into the phonological loop.^9^ Therefore, a listener who has difficulty accessing the phonological structure of language may demonstrate deficits on modality‐specific auditory tasks of verbal WM capacity, likely as a result of poorer encoding of the incoming signal into the memory buffer.

Two recent studies indirectly support the hypothesis that modality‐specific assessments of verbal WM are more relevant to speech recognition outcomes than modality‐general assessments. In a study of adult CI users by Tao et al., relations were identified between auditory measures of WM and sentence recognition in quiet and in speech‐shaped noise.^18^ In contrast, a recent study by Moberly, Houston, and Castellanos demonstrated no correlations for adult CI users between scores of sentence recognition in speech‐shaped noise and verbal WM scores for visual tasks of forward and reverse memory taken from the Leiter‐3 International Performance Scale,^19^ a well‐validated and widely used assessment of neurocognitive performance.^20^ Together, these findings suggest that verbal WM tested in a modality‐specific auditory fashion, as compared to a modality‐general visual fashion, contributes to sentence recognition abilities for CI users. However, neither of those two studies in CI users examined verbal WM in the same sample using both modality‐specific and modality‐general tasks. As a result, it remains unclear whether there are strong relations between verbal WM and sentence recognition in adult CI users, and whether these effects are limited to verbal WM tests that are modality‐specific, representing their ability to encode, store, and process auditory input, versus verbal WM tests that that are modality‐general, representing listeners' more foundational neurocognitive functions. For example, there is evidence from the pediatric literature that prelingual CI users demonstrate a number of deficits in neurocognitive processes, specifically executive functioning skills involving attention, WM, and inhibition,^21^ and in adult CI users, deficits in similar neurocognitive processes may result in problems with sentence recognition.

The first goal of the current study was to test the hypothesis that verbal WM performance using both a modality‐general visual task (RSpan) and a modality‐specific auditory task that is identical to RSpan except that stimuli are delivered auditorily instead of visually (Listening Span, LSpan) would predict sentence recognition scores in noise, both for adult CI users and normal‐hearing (NH) peers. It was further hypothesized that LSpan would predict sentence recognition more strongly than RSpan, as a result of the contribution of audibility factors during both the LSpan and sentence recognition tasks.

The second goal of this study was to test the hypothesis that more basic underlying neurocognitive skills would predict verbal WM performance on both the modality‐general task (RSpan) and the modality‐specific task (LSpan). During performance of any verbal WM task, a number of underlying modality‐general neurocognitive skills should come into play. In particular, these skills include the general capacity of WM, controlled fluency (the ability to process stimuli rapidly under concentration demands),^22^ and inhibition‐concentration (the ability to concentrate on information relevant to the task while suppressing prepotent or automatic responses not relevant to the task).^23^ In particular, support for the role of inhibition‐concentration in speech recognition comes from studies demonstrating that a reduction in older adults' ability to ignore task‐irrelevant information is an important contributor to their difficulty recognizing words in noise.^4^, ^24^, ^25^ Inhibitory processes may also facilitate the identification of correct lexical items and inhibit incorrect responses.^26^ Thus, the third goal of the current study was to test the hypothesis that modality‐general and modality‐specific tasks of verbal WM capacity, as measured using RSpan and LSpan, would mediate the effect of controlled fluency and/or inhibition‐concentration abilities on sentence recognition skills.

To accomplish the three goals of the current study, a group of postlingually deafened adult experienced CI users, along with a group of age‐matched peers with NH, were tested using a modality‐general task of verbal WM capacity (visual RSpan) and a modality‐specific task of verbal WM capacity (auditory LSpan). These measures were examined for their relations to previously reported measures of speech recognition and more basic neurocognitive functions,^20^ in order to provide further evidence that modality‐general neurocognitive functions underlie performance on both modality‐general and modality‐specific verbal WM tasks, which, in turn, contribute to sentence recognition skills for adult CI users and NH peers listening to speech in noise.

## MATERIALS AND METHODS

### Participants

Data from 60 adults were analyzed. Thirty participants were experienced CI users, between ages 50 and 82 years, who were recruited from the Otolaryngology department at The Ohio State University; 30 additional participants were age‐matched normal‐hearing (henceforth referred to as “NH”) controls. All participants underwent screening in order to ensure no evidence of cognitive impairment, normal general language proficiency, and normal near‐vision. Details of these participants can be found in the manuscript by Moberly, Houston, and Castellanos.^20^

### Equipment

All tasks were performed in a soundproof booth or a sound‐treated testing room as discussed in the Moberly, Houston, and Castellanos paper.^20^ Participants were tested using their usual devices (1 CI, 2 CIs, or CI plus contralateral hearing aid) or no devices (for NH controls). The experimenter checked all devices at the beginning of the testing session, and participants confirmed sound detection.

### Stimuli and stimuli‐specific procedures

#### Sentence Recognition

Three measures of recognition of words in sentences were included: 1) long, syntactically complex sentences (“long, complex” sentences); 2) short, meaningful, highly semantically constrained sentences (“short, meaningful” sentences); and 3) four‐word strings of words that were syntactically correct but semantically anomalous (“nonsense” sentences), and were described in detail in the manuscript by Moberly, Houston, and Castellanos.^20^

#### Verbal Working Memory

Computerized versions of a modality‐general task of verbal WM (RSpan) and an auditory modality‐specific task (LSpan) task were used, which are publicly available (http://www.millisecond.com). Participants saw on a computer monitor (RSpan) or heard over loudspeaker (LSpan) a number of sentences (unrelated to those used in the sentence recognition tasks). They stated whether each sentence made sense (true) or not (false), and were instructed to try their best to keep accuracy on the true/false judgment above 85% throughout the procedure. If the accuracy score for a participant on this true/false judgment was not above 85% throughout the procedure, a score of 0 was assigned for that participant for use in analyses. Following each true/false judgment, a single letter was presented on the computer monitor (RSpan) or over the speaker (LSpan). After presentation of a series of sentences, participants were asked to recall in correct sequential order the letters presented by clicking the corresponding letters on the computer screen. The total number of correct letters recalled in correct serial order served as the measure of interest.

#### Non‐Auditory Measures of Modality‐General Neurocognitive Functioning

Non‐auditory tasks from the Leiter‐3 International Performance Scale were used to assess controlled fluency and working memory.^19^ An additional non‐auditory computerized measure of verbally mediated inhibition‐concentration was also collected. Details of these neurocognitive measures can be found in the manuscript by Moberly, Houston, and Castellanos,^20^ and are described here in brief. Instructions for the Leiter‐3 tasks were given to participants through pantomime and gesturing, according to the Leiter‐3 manual. Raw scores were converted to standard scores, which were used in analyses.

##### Controlled Fluency

During the *Attention Sustained* task, participants were given 30 or 60 seconds to cross out as many figures as possible that matched target figures shown at the tops of pages of paper.

##### Working Memory

During *Forward Memory* and *Reverse Memory*, an easel was shown that demonstrated several pictures of animals in squares. The experimenter pointed to a sequence of pictures, and participants were required to point to the corresponding pictures in the same forward (or reverse) order.

##### Inhibition‐Concentration

A non‐auditory computerized version of a *Verbal Stroop* task was used, which has been made publicly available (http://www.millisecond.com). Participants saw color words one at a time on a computer screen and were required to name the color of the text of the word presented. Scoring was done automatically by the computer when the participant directly entered responses by pressing buttons that corresponded to the colors. Response times were computed for correct responses to “congruent” conditions (relying on implicit word reading; e.g., the word “Red” shown in red text) and to “incongruent” conditions (relying on inhibition of word reading and concentration on the text color; e.g., the word “Red” shown in blue text).

### General Procedures

Procedures were approved by The Ohio State University Institutional Review Board. Participants were tested in one session lasting approximately 2 hours. Two 10‐minute breaks were given during testing between task sessions to prevent fatigue. First, audiometric thresholds and screening measures were collected. All participants then completed sentence recognition testing, with randomization of different sentence materials presented in blocks and orders of sentences. Lastly, participants completed the tasks of verbal WM and non‐auditory neurocognitive functions, with task order randomized.

### Data Analyses

To test the first hypothesis, Pearson‐product bivariate correlation analyses were performed between scores on RSpan or LSpan and sentence recognition scores, for each group (CI and NH) separately. For the second hypothesis, correlation analyses were performed among more basic neurocognitive scores and RSpan and LSpan scores. To test the third hypothesis, linear regression models were performed to examine mediation effects of RSpan and LSpan on relations between basic neurocognitive scores and sentence recognition scores.

## RESULTS

Results from sentence recognition tasks and basic neurocognitive assessments are reported in Moberly, Houston, and Castellanos,^20^ and are repeated here in brief. Novel data from the RSpan and LSpan tasks, along with analyses for their relations with sentence recognition and basic neurocognitive assessments are reported below in detail.

First, for the CI group, side of implantation (left, right, or bilateral) did not influence any scores of sentence recognition, basic neurocognitive functions, RSpan, or LSpan (based on one‐way ANOVA results with *p* > .50). Also, no differences in any scores were found for CI users who wore only CIs versus a CI plus a contralateral hearing aid (based on independent‐samples *t*‐tests, *p* > .50). Therefore, the data were collapsed across all CI users in subsequent analyses. Scores for the sentence recognition tests were not normally distributed. Consequently, arcsine transformations were computed and used for subsequent analyses involving these tasks, with resulting scores normally distributed. Sentence recognition scores were not compared directly between CI and NH participant groups, because they were tested using different SNRs (see Table 1 for mean scores).

**Table 1:** Group mean modality‐general neurocognitive, modality‐specific auditory verbal working memory (LSpan), and sentence recognition scores and results of independent‐samples *t‐*tests. Sentence recognition scores were not compared between groups, because signal‐to‐noise ratio (SNR) was different between groups. For CI users, sentence recognition scores were presented at +3 dB SNR for long, complex and short, meaningful sentences and in quiet for nonsense sentences. For NH listeners, all sentence recognition tasks were presented at −3 dB SNR. CI = cochlear impant; LSpan = Listening Span; NH = normal hearing; RSpan = Reading Span; SD = standard deviation; SNR = signal‐to‐noise ratio.

	Groups							
	NH (*N* = 30)			CI (*N* = 30)				
	*N*	Mean	(SD)	*N*	Mean	(SD)	*t* value	*p* value
Sentence Recognition ‐ Long, complex (% words correct)	*30*	66.7	(14.4)	*30*	24.6	(22.4)		
Sentence Recognition ‐ Short, meaningful (% words correct)	*30*	81.7	(9.3)	*30*	40.5	(35.0)		
Sentence Recognition ‐ Nonsense (% words correct)	*30*	38.8	(11.7)	*30*	70.6	(19.0)		
Attention Sustained (scaled score)	*30*	10.2	(1.9)	*30*	9.6	(2.0)	1.20	.24
Forward Memory (scaled score)	*30*	13.0	(2.3)	*30*	11.8	(2.3)	2.08	**.04**
Reverse Memory (scaled score)	*30*	13.5	(2.4)	*30*	12.7	(2.2)	1.44	.16
Verbal Stroop–Congruent (response time in seconds)	*30*	1.22	(.30)	*28*	1.34	(.47)	1.15	.26
Verbal Stroop–Incongruent (response time in seconds)	*30*	1.57	(.47)	*28*	1.72	(.48)	1.16	.25
LSpan (total letters correctly recalled)	*30*	44.2	(12.3)	*25*	24.3	(20.3)	4.47	**<.001**
RSpan (total letters correctly recalled)	*30*	41.7	(12.9)	*30*	37.1	(17.9)	1.14	.259

The first hypothesis tested was that scores on the modality‐general task of verbal WM (RSpan) and the modality‐specific task of verbal WM (LSpan) would both correlate with sentence recognition scores in noise, for each group separately (CI and NH). For CI users, only LSpan scores correlated significantly with sentence recognition scores, for all 3 sentence types, as demonstrated in Table 2. RSpan scores did not correlate with any sentence recognition scores in CI users (*p* > 0.50 for all analyses). For NH controls, no significant correlations were identified between any of the sentence recognition scores and either LSpan or RSpan (*p* > 0.50 for all analyses). Thus, only scores on the modality‐specific measure of verbal WM (RSpan) correlated with sentence recognition in noise for CI users, and neither measure of verbal WM (RSpan and LSpan) correlated with sentence recognition in NH peers.

**Table 2:** *r* values from correlation analyses with recognition of words in sentences. CI users were tested at +3 dB SNR for long, complex and highly meaningful sentences, and in quiet for nonsense sentences. NH listeners were tested at −3 dB SNR for all sentence materials.

	Groups					
	NH			CI		
	Long, complex sentences	Highly meaningful sentences	Nonsense sentences	Long, complex sentences	Highly meaningful sentences	Nonsense sentences
**Verbal Working Memory**						
LSpan (total letters correctly recalled)	.10	.05	.07	.64**	.57**	.68**
Rspan (total letters correctly recalled)	‐.01	.06	.13	‐.03	.01	‐.02
						
**Modality‐General Neurocognitive Scores**						
Attention Sustained (scaled score)	.14	.07	‐.08	.14	.19	.29
Forward Memory (scaled score)	‐.10	‐.35	.17	.23	.23	.14
Reverse Memory (scaled score)	.06	‐.11	.08	.20	.20	.04
Verbal Stroop–Congruent (response time)	‐.04	.20	.07	‐.28	‐.29	‐.36
Verbal Stroop–Incongruent (response time)	‐.14	‐.05	‐.03	‐.41*	‐.43*	‐.43*
						
* *p*‐value < 0.05						
** *p*‐value < 0.01 CI = cochlear impant; LSpan = Listening Span; NH = normal hearing; RSpan = Reading Span						

The second hypothesis tested was that scores on the basic neurocognitive modality‐general tasks would correlate with verbal WM scores, both RSpan and LSpan, in both groups. For CI users, RSpan scores correlated with forward and reverse memory scores, as well as controlled fluency, as shown in Table 3; LSpan scores negatively correlated only with response times for the incongruent condition of the Stroop task. For NH controls, RSpan did not correlate with any of the basic neurocognitive measures; LSpan again negatively correlated only with response times for the incongruent condition of the Stroop task.

**Table 3:** *r* values from correlation analyses for working memory tasks and modality‐general neurocognitve scores for NH and CI participants.

	Groups			
	NH		CI	
	LSpan (total letters correctly recalled)	RSpan (total letters correctly recalled)	LSpan (total letters correctly recalled)	RSpan (total letters correctly recalled)
**Modality‐General Neurocognitive Scores**				
Attention Sustained (scaled score)	.14	‐.01	.04	.45*
Forward Memory (scaled score)	.16	.26	.16	.54*
Reverse Memory (scaled score)	.26	.03	.18	.42*
Verbal Stroop–Congruent (response time)	‐.28	.24	‐.26	‐.01
Verbal Stroop– Incongruent (response time)	‐.44*	.06	‐.59**	‐.27
				
* *p*‐value < 0.05				
** *p*‐value < 0.01 CI = cochlear impant; LSpan = Listening Span; NH = normal hearing; RSpan = Reading Span				

The third hypothesis tested was that our measures of modality‐general verbal WM (RSpan) and modality‐specific verbal WM (LSpan) would mediate the significant effects of inhibition‐concentration skills on sentence recognition ability.^20^ Specifically, for CI participants, only inhibition‐concentration was significantly associated with all 3 sentence recognition scores (*p* = .02‐.03 across the 3 sentence measures). In particular, response times measured during the “incongruent” condition correlated with sentence recognition scores; however, response times from the “congruent” condition did not. Thus, the speed of inhibitory control, but not general response speed, was associated with sentence recognition skills in CI users. On the other hand, for NH controls, none of the neurocognitive scores were associated with sentence recognition. As demonstrated above, RSpan scores did not correlate with sentence recognition scores for either CI or NH participants. Moreover, LSpan did not correlate with sentence recognition in NH listeners. For these reasons, RSpan could not serve as a mediator of inhibition‐concentration on sentence recognition for either group, nor could LSpan serve as a mediator for NH participants. Thus, a mediation model was tested only for CI participants.

To test the prediction that LSpan would mediate the effects of inhibition‐concentration on sentence recognition in CI users, 3 separate sets of regression analyses were conducted for each of the sentence recognition assessments. We followed Baron and Kenny's model for testing mediating effects.^27^ The first set of regression equations tested for direct relations between each of the neurocognitive skills and sentence recognition performance. The second set of regression equations tested for direct relations between each of the neurocognitive skills and LSpan. Finally, the third regression equation tested the full mediating model with simultaneous entry of neurocognitive skills and LSpan as predictors of sentence recognition.

**Figure 1:**
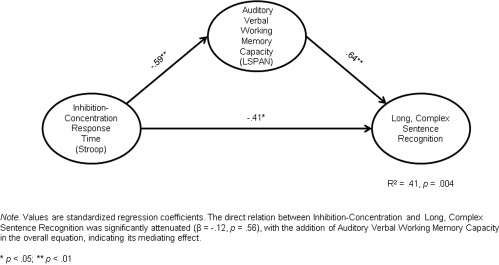
Mediating model predicting recognition of words in long, complex sentences in CI users. CI = cochlear implant

**Figure 2:**
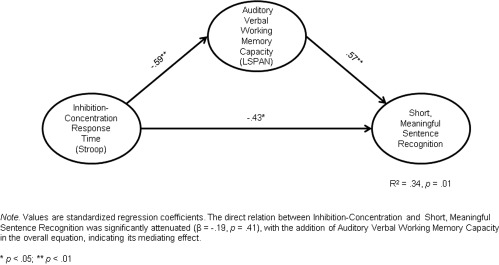
Mediating model predicting recognition of words in short, meaningful sentences in CI users. CI = cochlear implant

**Figure 3:**
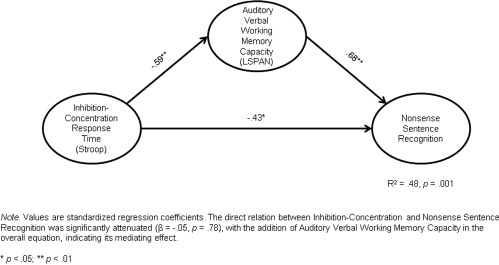
Mediating model predicting recognition of words in nonsense sentences in CI users. CI = cochlear impant

The direct relations between inhibition‐concentration (incongruent condition of Stroop) and all three sentence recognition scores were significant (long, complex sentences, β = ‐.41, *p* = .03; short, meaningful sentences, β = ‐.43, *p* = .02; and nonsense sentences, β = ‐.43, *p* = .02), independent of any mediating effects. The direct relation between inhibition‐concentration response times and LSpan was also significant (β = ‐.59, *p* = .002). The overall equations revealed that LSpan mediated the effects of inhibition‐concentration on all 3 sentence recognition scores (see Figs. 1– 3). This was evident because the direct relations between inhibition‐concentration and all three sentence recognition scores were no longer significant with the addition of LSpan in the overall equation (all *p* > .10), indicating the mediating effects of the modality‐specific measure of verbal WM.

## DISCUSSION

This study examined whether verbal WM of postlingually deafened adult CI users would be associated with the ability to recognize words in sentences, and if this relation would be dependent on whether verbal WM was assessed using a modality‐general (visual RSpan) task or if the relation would only exist when verbal WM was assessed using a modality‐specific (auditory LSpan) task. Moreover, the study sought to identify the contributions of more basic modality‐general neurocognitive factors to verbal WM. Lastly, the study sought to investigate whether verbal WM would serve to mediate the effects of basic neurocognitive functions on the abilities of CI users and NH peers to recognize sentences in noise. The study was motivated by previous findings suggesting that WM declines underlie variability in outcomes in adults with CIs. For example, Bhargava, Gaudrain, and Başkent identified poorer intelligibility of interrupted meaningful sentences in adult CI users than in NH listeners, and attributed poorer performance at least in part to older age in the CI users.^28^ Although not specifically mentioning WM, the authors of that study suggest a general slowing of cognitive processing in older CI users may have deleteriously affected their use of top‐down mechanisms during speech recognition. A study by Tao et al. found relations between auditory measures of verbal WM (using forward and reverse digit span) and sentence recognition in quiet and in speech‐shaped noise; however, findings of that study were confounded by the issue of audibility during the WM tasks.^18^

The first finding of this study was that verbal WM correlated with sentence recognition skills, but only for adult CI users, and only when using scores from the modality‐specific LSpan task of verbal WM. In contrast to previous findings of RSpan as a robust predictor of speech recognition in adults listening under degraded conditions, we failed to find any significant relations between RSpan scores and speech recognition scores for either the CI users or NH listeners. This lack of relationship might be attributable to the fact that our listeners were tested in speech‐shaped noise, which essentially provides energetic masking; stronger relations between RSpan and speech recognition skills have been found when listeners are presented speech in modulated noise or with informational masking.^29^ Second, our computerized RSpan task asked participants to recall single letters presented after sentences. As a result, the items to be stored and recalled may not have been encoded phonologically. For CI users, being able to recognize words appears to be highly dependent on access to the phonological structure of the speech.^30^ Nonetheless, our failure to find a relation between scores on the RSpan task and sentence recognition for either CI users or NH controls suggests that WM capacity as measured under relatively ideal sensory conditions is not related to recognition of speech under degraded conditions. On the other hand, LSpan did significantly predict sentence recognition in CI users. Findings suggest that variability in CI users' ability to encode into WM verbal information that is delivered auditorily contributes to speech recognition performance.

The second hypothesis tested was that modality‐general basic neurocognitive skills, assessed using non‐auditory tasks, would predict performance on the RSpan and LSpan tasks of verbal WM. This hypothesis was partially supported by data from both CI users and NH controls indicating that scores on the inhibition‐concentration task were associated with better LSpan performance. In other words, modality‐general inhibition‐concentration skills contribute to verbal WM ability, consistent with existing models of WM,^9^, ^31^ but this relation was only found for LSpan. Interestingly, the non‐auditory measures of forward and reverse memory and controlled fluency were associated with RSpan scores for CI users but not with LSpan, and these measures did not correlate with either RSpan or LSpan for NH controls. These findings may again represent limitations in the RSpan task, as previously discussed, or may suggest that the tasks we used from the Leiter‐3 (*Attention Sustained* and *Forward* and *Reverse Memory*) are not robust measures at identifying the neurocognitive abilities that underlie language processing.

The third hypothesis tested was that verbal WM would mediate the effects of inhibition‐concentration abilities on sentence recognition. Our results partially supported this hypothesis: LSpan scores fully mediated the effects of inhibition‐concentration on sentence recognition, but RSpan did not. These findings provide support for a central role of modality‐specific verbal WM in the process of speech recognition for CI users. This is consistent with the Ease of Language Understanding (ELU) model, which suggests that under degraded listening conditions, successful speech perception requires a degree of effortful, controlled processing, which is heavily dependent on verbal WM.^5^ Results are also consistent with findings by Sommers and Danielson,^26^ who identified individual differences in inhibitory control as contributing to sentence recognition performance in adults with NH, consistent with models of speech perception that consider the need for a listener to inhibit interference of irrelevant information, or to inhibit prepotent but incorrect responses.^32^ However, in contrast to the findings of Sommers and Danielson,^26^ we did not find significant relations between inhibition‐concentration skills and sentence recognition in NH listeners. This lack of a relationship could be a result of the relatively narrow range of performance demonstrated by the NH participants on sentence recognition, or it could suggest a differential relation between inhibition‐concentration processes and sentence recognition between CI and NH listeners. In support of the latter idea, a recent study by Füllgrabe & Rosen demonstrated that weaknesses in verbal WM contributed to difficulties in recognizing words in sentences for CI users, but not for NH controls.^15^ Further studies will be required to better understand these differential relations between groups (CI and NH) and why verbal WM appears to account for the relation between inhibition‐concentration and sentence recognition only among CI users.

Results of the current study have two major clinical implications. First, findings provide further support for the idea that the modality‐general neurocognitive skills of inhibition‐concentration contribute to speech recognition outcomes in adult CI users, as do verbal WM skills, at least when assessed using an auditory task. As a result, it is evident that we should provide greater research and clinical emphasis on top‐down processing for CI users to optimize speech recognition outcomes. Second, there may be a benefit to addressing inhibition‐concentration and verbal WM skills through novel clinical aural rehabilitation programs.

## CONCLUSION

Our findings indicate that modality‐general inhibition‐concentration skills contribute to CI users' abilities to recognize words in sentences. This effect was fully explained by differences in modality‐specific verbal WM skills. Findings provide further evidence for the role of top‐down processing by CI users and imply potential benefits of developing clinical aural rehabilitation programs that target inhibition and verbal WM skills.
